# Could Nodding Syndrome (NS) in Northern Uganda be an environmentally induced alteration of ancestral microbiota?

**DOI:** 10.11604/pamj.2018.31.152.14142

**Published:** 2018-10-30

**Authors:** Denis Anywar Arony, Suzanne Gazda, David Lagoro Kitara

**Affiliations:** 1 Gulu University, Faculty of Medicine, Department of Biochemistry, Gulu, Uganda; 2 Founding President of Hope for Humans (HfH) and Neurologist, San Antonio, Texas, USA; 3 Gulu University, Faculty of Medicine, Department of Surgery, Gulu, Uganda

**Keywords:** Nodding syndrome, internal displacement, food ration, microbiota-gut-brain axis

## Abstract

Hippocrates stated in 460-C.370 BC that, “All diseases begin in the Gut.” This statement may be beginning to have meanings in the advent of new diseases such as Nodding Syndrome (NS) and Autism Spectrum Disorder (ASD). Interestingly, a recent publication from China in the journal of microbiology in 2017 suggests that high grain diet had dynamically shifted the composition of mucosa-associated microbiota and induced mucosal Injuries in the colon of Sheep. NS is a devastating childhood neurological disorder characterized by atonic seizure, cognitive impairment, head nodding, wasting and stunted growth. In addition, NS in Northern Uganda is clustered in time (those who were in IDPs), in space (discretely observed on either sides of the two rivers of Aswa and Pager) and in person (onset mainly between the ages of 5-15 years) and therefore exhibits spatial temporality. The first case of NS was noticed in Kitgum district in 1997, one year after the reported displacement of that community into IDP. Prior to that internal displacement, there were no reported cases of NS. The same scenario occurred in the IDPs of Odek, Gulu district where the population was displaced into IDPs in 2001 and approximately a year later in 2002, cases of NS began to appear. In the IDPs, children that eventually developed NS fed nearly exclusively on food ration provided by relief agencies and roughly a year later, cases of NS began to appear. In the other East African countries, there were no reported cases of NS prior to internal displacement and dependence on food ration. The observed common factors in the three East African regions where NS occurs at endemic proportion are perhaps: Internal displacement and feeding on relief food. These researchers suggest that NS may have perhaps resulted from dietary and environmental factors during IDPs which may have been foreign to their GIT and links this observation to the concept of microbiota-gut-brain axis.

## To the editors of the Pan African Medical Journal

Nodding Syndrome (NS) is a devastating neurological disorder which begins in children that are subject to civil disruption, internal displacement and feeding on foods foreign to their microbiota [1]. Interestingly, there have been extensive searches for infectious causes of NS in the region with no uniform identifiable link [2-4]. It is reported in many studies that children that developed NS were born normal with normal developmental milestones and that upon developing NS which was mainly observed in children who were subject to internal displacement and fed on food rations, NS began to appear [1-3]. The foods eaten before onset of NS were supplementary and weaning foods e.g. cereals/grains, powdered milk, beans, soya, yellow posho and cooking oil [1]. Most of these food items eaten during the IDPs were reportedly not usual diet to NS children and perhaps foreign to their microbiota [1, 3]. Moreover, there were multiple reports that these food were sometimes spoiled/rotten or rather tasted bitter [1-3]. The question is why has it mainly occurred in children? Many researchers have suggested that children are more vulnerable in such a circumstance because of greater exposure-pound-for-pound; their decreased ability to detoxify many toxins/chemicals; heightened biological vulnerability (e.g. Thalidomide and fetal alcoholic syndrome) and many years of future life (Table 1). It is reported that IDP situations was associated with malnutrition, social norm breakdown, mental health disorders, increasing prevalence of infectious diseases, neglect and waste of the youths [1-4]. In addition, there is a widely held belief among communities in Northern Uganda that NS had possibly originated from contaminated relief food eaten in IDPs or exposure to war munitions/chemicals during the war [4-6]. Similarly, the peaks of reported NS onset also correlated with peaks of household displacement and dependence on food aid provided by the relief agencies [1-3]. These IDP camps were reportedly insecure, unsanitary, squalid, overcrowded with food insecurity and high potential for disease transmission [4, 5].

**Table 1:** The possible causes of leaky gut that may have occurred in NS children

Toxic and inflammatory foods	Medications
Alcohol	Antibiotics
Diary	NSAIDs (Aspirin, Ibuprofen)
Eggs	Birth control pills
Gluten	Prednisolone
Grains and pseudograins	Acid blocking drugs
Legumes	**Chemotherapy**
Genetically Modified foods (GMO)	**Radiation**
Nightshades	**Surgeries**
Sugar	**Myocotoxins (toxic molds)**
**Gut Infections**	**Stress**
SIBO	Emotional stress
Candida	Physical Stress
Parasites	

A neurologist, Dr Gazda wrote, “You are the first to ever put this into the equation, the many factors that were at play in the epidemiology of NS (Table 1). The local culture and history of this region was drastically changed by the long horrific war, the timeline of the impact of the multiple stressors and overall the appalling conditions of the IDP camps. This information has been it seems, the *unspeakable truth* or perhaps we should say as Al Gore phrased it, *the inconvenient truth*. There is no doubt in my mind after observing these NS children for over 5 years that, NS is a mitochondrial disease that was environmentally induced by multiple factors that resulted in the *perfect storm* in perhaps a genetically predisposed population naive and previously immune to such risk factors“ (Table 1). She added, “Hippocrates was right when he said in 460-c. 370 BC that, *all diseases begin in the gut* and in this population; it was all the above factors and more that altered the microbiome which is passed down at birth from their mothers and considered *our ancient friends* and because our microbiome is the control center of health and allows us to be healthy, it resulted in epigenetic changes and this neurodevelopmental disease we now call Nodding Syndrome. These NS children perhaps have a mitochondrial environmentally induced Autism Spectrum Disorder.” She further added, “It was the imperfect storm that through accumulation of factors, led to this problem. NS is perhaps an environmentally induced neurodevelopmental disease which is much more than just epilepsy.”

Another Neuroscientist, Professor Spencer said, “Your data on NS adds some useful information to the historical, environmental and nutritional data we have published previously [2]. The statements you made regarding immunization coverage stand in marked contrast to several documents we have cited about the April 1999 UN report about IDPs in Masindi district in Western Uganda [2]. A recent visit to Masindi by UNICEF and AVSI found during a Polio campaign that, there had been no immunization of IDP children from 1996-1999.” The suggestion by Prof. Spencer is very informative in support of this theory. It shows that whereas some of the Acholi population was displaced in different locations within Uganda, there were no reported cases of NS in those internally displaced communities except those in IDPs of Northern Uganda. In Masindi district and elsewhere, IDPs fed on their own home grown foods in sharp contrast to the same population displaced within Northern Uganda who were nearly exclusively fed on food ration provided by the relief agencies. Interestingly, to-date there are no reported cases of NS among children born and raised in Masindi district and those in the LRA captivity yet they too were displaced. The difference perhaps was that they did not majorly feed on the similar food rations provided by the relief agencies. In addition, children born and raised in LRA captivity reportedly had no or had minimal access to vaccinations while in the bush, a factor that perhaps eliminates the lack of vaccination as the possible aetiological factors in the onset of NS. This finding perhaps raises the issue that the aetiology of NS may be more of dietary and environmental factors that was peculiar and experienced by NS children displaced and raised in specific IDP camps within Northern Uganda.

**Microbiota-gut-brain axis:** studies have shown that there is a communication between the gut and brain called the microbiota-gut-brain axis [7]. Recently, there is growing evidence that the microbiota that resides in the gut can modulate brain development and produce behavioral phenotypes via this axis [8]. This bidirectional communication acts mainly through neuroendocrine and neuroimmune mechanisms [8]. More studies have shown that gut microbiota participates in cerebral disorders by modulating immune responses of its host by stimulating the secretion of pro-inflammatory cytokines (including IL-1, IL-6 and IL-18) by intestinal epithelial cells, intestinal dendritic cells and macrophages [9]. These observations suggest that pathogenic microbiota, bacterial metabolites and their components are able to increase levels of circulating cytokines closely associated with various neuropsychiatric disorders, including depression, anxiety, schizophrenia and ASD [10].

## Conclusion

Nodding Syndrome in Northern Uganda is a devastating childhood neurological disorder that may perhaps be a disease resulting from dietary and environmental factors experienced during the internal displacement into the IDPs in specific locations within Northern Uganda. It's a high time researchers began to focus their attention to environmental and dietary factors in search for the cause(s) of Nodding Syndrome. In addition, it may not be farfetched to begin thinking of microbiota-gut-brain axis as a possible aetiological factor in the pathogenesis of Nodding Syndrome in Northern Uganda.

## Competing interests

The authors declare no competing interests.

## References

[1] Denis Anywar Arony, Collines Angwech, Edward Frederick Makumbi, Suzanne Gazda, David Lagoro Kitara (2017). Is there a line between internal displacement; environmental and dietary factors in the onset of Nodding Syndrome in northern Uganda. A clinical observational study design.

[2] Spencer PS, Mazumder R, Valerie SP, Lasarev MR, Stadnik RC, King P, Kabahenda M, Kitara DL, Stadler D, McArdle B, Tumwine JK (2016). other Members of the Oregon-Uganda Nodding Syndrome Research Team. Environmental, dietary and case-control study of Nodding Syndrome in Uganda: a post-measles brain disorder triggered by malnutrition. J Neurol Sci.

[3] Kitara DL, Anywar AD, Mwaka AD, Uwonda G, Abwang B, Kigonya E (2013). Nodding syndrome in Northern Uganda: a probable metabolic disorder. Br J Med Med Res.

[4] Landis JL, Palmer VS, Spencer PS (2014). Nodding syndrome in Kitgum District, Uganda: association with conflict and internal displacement. BMJ Open.

[5] World Food Program Food Security Assessment of IDP Camps in Gulu, Kitgum, and Pader Districts, October 2006.

[6] Kitara DL, Amone C (2012). Perception of the Population in Northern Uganda to Nodding Syndrome. J Med Med Sci.

[7] Sanger GJ, Lee K (2008). Hormones of the gut-brain axis as targets for the treatment of upper gastrointestinal disorders. Nat Rev Drug Discov.

[8] Diaz HR, Wang S, Anuar F, Qian Y, Bjorkholm B, amuelsson A, Hibberd ML, Forssberg H, Pettersson S (2011). Normal gut microbiota modulates brain development and behavior. Proc Natl Acad Sci USA.

[9] Maynard CL, Elson CO, Hatton RD, Weaver CT (2012). Reciprocal interactions of the intestinal microbiota and immune system. Nature.

[10] Petra AI, Panagiotidou S, Hatziagelaki E, Stewart JM, Conti P, Theoharides TC (2015). Gut-microbiota-brain axis and its effect on neuropsychiatric disorders with suspected immune dysregulation. Clin Ther.

